# Early Chronic Pancreatitis Findings by Endoscopic Ultrasonography (EUS) in Asymptomatic Patients with Pancreas Divisum

**DOI:** 10.3390/diagnostics15030253

**Published:** 2025-01-22

**Authors:** Masatsugu Nagahama, Yuichi Takano, Fumitaka Niiya, Naoki Tamai, Jun Noda, Masataka Yamawaki, Tetushi Azami

**Affiliations:** Division of Gastroenterology, Department of Internal Medicine, Showa University Fujigaoka Hospital, 1-30 Fujigaoka, Aoba-ku, Yokohama 227-8501, Kanagawa, Japan; yuichitakano1028@yahoo.co.jp (Y.T.); spice_up_yourlife_10_4@yahoo.co.jp (F.N.); tama.226st.hope@gmail.com (N.T.); nodaji0317@gmail.com (J.N.); masataka-ymwk@outlook.jp (M.Y.); azazabc@icloud.com (T.A.)

**Keywords:** early chronic pancreatitis, pancreas divisum, PD, ECP, EUS

## Abstract

**Background/Objectives:** Pancreas divisum (PD) is a congenital malformation associated with chronic and recurrent acute pancreatitis. Although PD often presents asymptomatically, the extent to which early chronic pancreatitis (ECP) changes occur in asymptomatic patients with PD remains unclear. This study aimed to evaluate endoscopic ultrasonography (EUS) findings indicative of ECP in asymptomatic patients with PD and investigate the relationship between these findings and background factors, such as age, sex, main pancreatic duct diameter, and alcohol intake. **Methods:** We retrospectively analyzed 17 asymptomatic patients diagnosed with PD at the Showa University Fujigaoka Hospital between January 2016 and May 2024. EUS was used to assess the pancreatic parenchyma and ductal features, and the findings were classified according to the Rosemont Classification (RC) and the Japan Pancreas Society (JPS) 2019 criteria for ECP (JDCECP2019). Statistical analyses were performed to examine the association between EUS findings and patient background factors. **Results:** EUS findings of ECP were observed in 8 of 17 asymptomatic patients with PD (47%) according to both the RC and the JDCECP2019 criteria. Hyperechoic foci without shadowing or strands were the most common findings, present in 82% of the patients. No significant associations were found between EUS findings and the background factors of age, sex, main pancreatic duct diameter, or alcohol intake. **Conclusions:** A considerable proportion of asymptomatic patients with PD exhibited EUS findings suggestive of early chronic pancreatitis. These findings indicate that early changes in chronic pancreatitis may be accompanied by asymptomatic PD.

## 1. Introduction

Pancreas divisum (PD) is a potential cause of chronic and recurrent acute pancreatitis [1,2,3,4]. The main clinical symptoms associated with PD include recurrent acute pancreatitis, chronic pancreatitis, and chronic abdominal pain, but these occur in only 5% of the patients with PD [5]. Although many patients with PD are asymptomatic, the extent of chronic pancreatitis changes in asymptomatic patients with PD has not been thoroughly investigated.

Recently, a novel concept and definition of chronic pancreatitis, termed the mechanistic definition, has been proposed [6]. According to the conceptual model of chronic pancreatitis based on the mechanistic definition, patients with chronic pancreatitis are categorized into one of five stages: at risk, acute pancreatitis–recurrent acute pancreatitis (AP-RAP), early chronic pancreatitis (ECP), established chronic pancreatitis (CP), and end-stage CP. Under this framework, the anatomical structure of PD is considered to correspond to the at-risk stage. The stages of AP-RAP and ECP are thought to precede symptomatic PD. However, diagnosing ECP remains clinically challenging due to the lack of sensitive and specific methods or a gold standard among the available techniques. Masamune et al. [7] conducted a two-year follow-up study of 83 patients diagnosed with early chronic pancreatitis (ECP). They reported that four (4.8%) patients progressed to definite chronic pancreatitis. However, the diagnosis of 48 (57.8%) patients was unchanged, and that of 31 (37.3%) patients was downgraded. These findings suggest that many cases of ECP may not progress to overt pancreatitis.

Endoscopic ultrasonography (EUS) is a well-established diagnostic modality that provides detailed examination and scoring of the pancreatic parenchyma and ducts, owing to its high resolution and ability to closely observe the pancreas [8]. Previous studies have demonstrated the superior sensitivity of EUS compared to ERCP [9,10,11,12]. Therefore, EUS may be capable of identifying subtle changes indicative of early chronic pancreatitis (ECP) in patients [8,13,14]. Early diagnosis of changes in chronic pancreatitis in asymptomatic patients with PD could enable preventive interventions before symptoms appear.

## 2. Materials and Methods

### 2.1. Patients

Among the cases diagnosed with PD dilatation via EUS and MRCP at Showa University Fujigaoka Hospital between 1 January 2016 and 25 May 2024, PD without a history of pancreatitis and without concomitant ampullary or pancreatic tumors was defined as asymptomatic PD and was included in this study.

### 2.2. Diagnosis of PD by MRCP Findings [15,16]

PD was diagnosed when the dorsal pancreatic duct crossed the common bile duct, drained into the duodenum via the minor papilla, and separated from the smaller ventral duct on MRCP images.

### 2.3. Diagnosis of PD by EUS Findings [17,18]

In this study, patients were diagnosed with PD based on any of the following EUS findings:

1. Crossed duct sign: EUS observation from the duodenal bulb shows the main pancreatic duct crossing the bile duct and extending to the minor papilla.
2. Absence of the stack sign: the common bile duct and pancreatic duct do not appear parallel when viewed from the duodenum.
3. Pancreatic duct not crossing the ventral–dorsal transition: the pancreatic duct does not cross the transition between the ventral and the dorsal pancreatic segments.

### 2.4. Endoscopic Ultrasonography (EUS)

EUS was performed using a curved linear array scope with frequencies of 6–7.5 MHz (GF-UCT260 endoscope [Olympus Medical Systems, Tokyo, Japan] and a UE-ME1 or UE-ME2 observation device [Olympus Medical Systems, Tokyo, Japan]). All patients were placed in the left lateral decubitus position. Analgesics and sedatives (pethidine hydrochloride [35 mg] or pentazocine [7.5–15 mg] plus midazolam [1.0–5.0 mg]) were administered during the procedure.

### 2.5. EUS Image Analysis

All EUS images obtained during the procedure were stored electronically. EUS findings related to ECP in these images were retrospectively confirmed and analyzed through discussions between two endoscopists (Masatsugu Nagahama and Yuichi Takano) with more than 10 years of experience in EUS examinations and a board-certified trainer at the Japan Gastroenterological Endoscopy Society.

The main pancreatic duct (MPD) of each patient was measured as the maximum diameter of the pancreatic body on the EUS images. The obtained EUS images were classified according to the Rosemont Classification (RC) [19] and the Japan Pancreas Society (JPS) proposed diagnostic criteria for ECP (JDCECP 2019) [20].

The RC classifies EUS features into major and minor categories, depending on their relevance to diagnosis. The major features are further subdivided into major A and major B.

The major features of A include hyperechoic foci with shadowing within the parenchyma and duct calculi with acoustic shadowing. The major B feature is lobularity (referred to as “honeycombing”) of the parenchyma. The minor features of the Rosemont criteria include hyperechoic foci without shadowing, hyperechoic strands, lobularity, and cysts of the parenchyma. Additional minor features include dilated duct, irregular duct contour, hyperechoic duct wall, and dilated side branches. Based on these endoscopic features, each case was classified as consistent with chronic pancreatitis (CP), suggestive of CP, indeterminate for CP, or normal. A diagnosis of “consistent with CP” requires one major A feature and three or more minor features, one major A feature and one major B feature, or two major A features. A diagnosis of “suggestive of CP” is based on one major A feature and three minor features, one major B feature and three or more minor features, or five or more minor features. “Indeterminate for CP” is diagnosed with three to four minor features, a major B feature alone, or a major B feature with three or fewer minor features. “Normal” was defined as having two or fewer minor features or no major features, excluding cysts, dilated MPD, hyperechoic foci without shadowing, and dilated side branches.

The Rosemont Classification of endoscopic ultrasound findings is defined as follows:

(1) Parenchymal criteria:

- Hyperechoic foci with shadowing: >2 mm in length/width with shadowing within the parenchyma.
- Hyperechoic foci without shadowing: >2 mm in length/width, without shadowing.
- Stranding: ≥3 mm in at least 2 different directions with respect to the imaged plane.
- Lobularity with honeycombing: ≥3 contiguous lobules = “honeycombing”.
- Lobularity without honeycombing: >5 mm, noncontiguous lobules.
- Hyperechoic main pancreatic duct margin: echogenic, distinct structure >50% of the entire main pancreatic duct in the body and tail.

(2) Ductal criteria:

- Dilated side branches: >3 tubular anechoic structures, each measuring ≥1 mm in width, budding from the main pancreatic duct.
- Cyst: anechoic, round/elliptical with or without septations.
- Main pancreatic duct dilatation: ≥3.5 mm in body or >1.5 mm in tail.
- Main pancreatic duct calculi: echogenic structures within the main pancreatic duct with acoustic shadowing.
- Irregular main pancreatic duct contour: uneven or irregular outline and ectatic course.

The EUS findings according to JDCECP 2019 were as follows: (1) hyperechoic foci (non-shadowing) or strands; (2) lobularity; (3) hyperechoic MPD margin; (4) dilated side branches. At least two of the four EUS findings, including either (1) or (2), are required for the diagnosis of ECP [21,22]. We investigated the frequency of EUS findings for chronic pancreatitis as defined by the RC and the JDCECP in the entire cohort. Additionally, we examined the number of cases diagnosed with CP based on the RC and those diagnosed with early chronic pancreatitis based on the EUS findings defined by the JDCECP 2019.

Furthermore, we investigated the relationship between ECP-related changes and findings in asymptomatic patients with PD, as well as background factors (age, sex, MPD diameter, and alcohol intake).

### 2.6. Statistical Analysis

Continuous variables are expressed as medians. The incidence and concordance between the two groups were compared using Fisher’s exact test and the Mann–Whitney U test, as appropriate. *p*-values < 0.05 were considered statistically significant. Data were analyzed using JMP Pro version 17.0.0., developed by SAS Institute Inc. (Cary, NC, USA).

### 2.7. Ethical Approval and Consent to Participate

All procedures were performed in accordance with the ethical standards of the 1964 Declaration of Helsinki. The study protocol was approved by the Institutional Ethics Committee of Showa University, Japan (IRB approval number: 2024-085-B; IRB approval date: 3 July 2024). All patients included in this study provided informed consent for the use of anonymous data using an opt-out methodology.

## 3. Results

**Figure 1:**
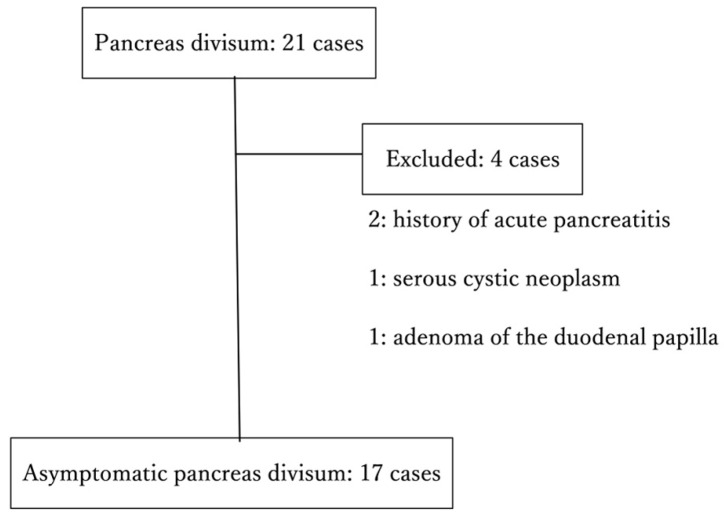
Cases of asymptomatic pancreas divisum.

Between 1 January 2016 and 25 May 2024, a total of 16,403 MRCP examinations were performed at Showa University Fujigaoka Hospital, out of which 87 cases were diagnosed with pancreas divisum (PD) based on MRCP findings. Among these, EUS was performed in 50 cases, and PD was confirmed by EUS in 21 cases. Of these individuals, 17 were included in this study after excluding 4 patients: 2 with a history of acute pancreatitis, 1 with a serous cystic neoplasm (SCN), and 1 with an adenoma of the duodenal papilla (Figure 1). The clinical characteristics of the 17 patients with asymptomatic PD are shown in Table 1.

The EUS findings according to the RC are shown in Table 2. Hyperechoic foci without shadowing were observed in 9 of the 17 patients (53%), and stranding was observed in 11 of the 17 patients (65%).

**Figure 2:**
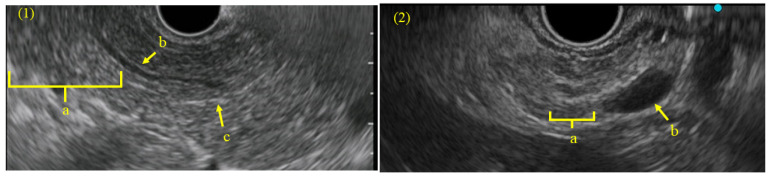
EUS findings according to the Rosemont Classification. (**1**) a. Hyperechoic foci without shadowing. b. Hyperechoic MPD margins. c. Stranding. (**2**) a. Lobularity without honeycombing. b. cyst. EUS: endoscopic ultrasonography, MPD: main pancreatic duct.

Lobularity without honeycombing was observed in 3/17 patients (18%), hyperechoic MPD margins in 5/17 (29%), dilated side branches in 7/17 (41%), and cysts in 6/17 (35%) (Figure 2(1),(2)). According to the RC, the EUS findings were categorized into major and minor criteria; however, in this study, asymptomatic patients with PD showed no EUS findings consistent with the major criteria and only presented with minor criteria. According to the RC, three patients (18%) were classified as having findings suggestive of chronic pancreatitis, and five (29%) were classified as having findings indeterminate for chronic pancreatitis.

The EUS findings according to the JPS diagnostic criteria for ECP are shown in Table 3. Hyperechoic foci [non-shadowing] or strands were observed in 14/17 patients (82%), lobularity was observed in 3/17 patients (18%), hyperechoic MPD margins were observed in 5/17 patients (29%), dilated side branches were observed in 7/17 patients (41%), and cysts were observed in 6/17 patients (35%). According to the ECP diagnostic criteria established by the JDCECP 2019, eight patients (47%) were diagnosed with EUS findings of ECP.

The diagnoses of the eight patients diagnosed with findings suggestive of chronic pancreatitis and of those who presented indeterminate findings according to the RC were consistent with the diagnoses based on EUS findings of ECP according to the JDCECP 2019. Additionally, a comparative analysis of the background factors in the eight patients diagnosed with suggestive chronic pancreatitis or indeterminate findings according to the RC and those of the nine patients with normal findings was conducted (Table 4). There were no statistically significant differences in sex distribution (*p* = 0.64), age (median: 63 vs. 59 years, *p* = 0.70), or main pancreatic duct (MPD) diameter (median: 2.15 mm vs. 2.2 mm, *p* = 0.70). The proportion of alcohol users was also comparable between the groups (50% vs. 44%, *p* = 1.00).

Furthermore, a comparative analysis of the EUS findings according to the RC based on the presence or absence of a history of alcohol consumption was conducted (Table 5). Among alcohol users and non-users, there were no significant differences in sex distribution (*p* = 0.64), age (median: 63 vs. 71 years, *p* = 0.29), MPD diameter (median: 2.6 mm vs. 2.3 mm, *p* = 0.50), or the number of minor criteria (median: 2.5 vs. 2, *p* = 0.62). No major criteria (A or B) were identified in either group. The EUS-based chronic pancreatitis (CP) diagnoses did not differ significantly between the groups for “suggestive” (*p* = 1.00) or “indeterminate” (*p* = 0.62) classifications.

## 4. Discussion

PD is a congenital malformation of the pancreatic ductal anatomy, in which the two distinct dorsal (Santorini duct) and ventral ducts (Wirsung duct) fail to fuse [23,24,25,26]. It has been suggested that the opening of the dorsal duct into the relatively smaller minor papilla increases intraductal pressure, potentially impairs the drainage of pancreatic secretions, and causes inflammation [27]. In recent years, the diagnosis of PD has become more common because of the detailed examination of pancreatic abnormalities, such as cystic lesions, pancreatic duct dilatation, and hyperechoic lesions, using abdominal ultrasound (AUS), computed tomography (CT), MRCP, and other imaging modalities during medical checkups and health examinations. The gold standard for diagnosing PD is direct contrast imaging of the pancreatic duct using endoscopic retrograde pancreatography (ERP) [28]. However, a limitation of ERP is the risk of inadvertent pancreatitis [5,28]. Recently, MRCP and EUS have emerged as viable alternatives for diagnosing PD, with reports suggesting that EUS has a higher diagnostic rate than MRCP [15,29]. In this study, patients with any of the following EUS findings were diagnosed with PD: (1) crossed duct sign; (2) absence of stack sign; (3) pancreatic duct not crossing the ventral–dorsal transition. These findings are important features of EUS for the diagnosis of PD [17]. PD is known to cause chronic and recurrent acute pancreatitis; however, whether changes in chronic pancreatitis occur in asymptomatic patients with PD remains unclear. In this study, hyperechoic foci (non-shadowing) or strands, as defined by RC, were observed in 14 of 17 asymptomatic patients with PD. Moreover, eight patients (two diagnosed with suggestive chronic pancreatitis and six with indeterminate chronic pancreatitis according to the RC) were also diagnosed with EUS findings of ECP by the JDCECP 2019. Thus, ECP changes were observed in 8 of 17 asymptomatic patients with PD (47%), according to both the RC and the JDCECP 2019. Although the RC does not define ECP, findings suggestive of or indeterminate for chronic pancreatitis based on EUS findings were considered to correspond to early chronic pancreatitis, according to the JDCECP 2019. These findings suggest that changes in ECP may occur at a certain frequency in asymptomatic patients with PD. We compared the presence or absence of EUS findings of ECP with respect to background factors, such as age, sex, MPD diameter, and alcohol intake, but found no significant differences. Additionally, when comparing the EUS findings according to the RC in relation to alcohol intake, no significant differences in background factors or EUS findings were observed. Yadav D. et al. [30] showed that a significant increase in the risk of chronic pancreatitis occurs only above a threshold of ≥five alcoholic drinks per day. Yamabe et al. [31] showed that three risk factors other than age (ethanol intake, smoking, and history of acute pancreatitis) were positively correlated with RC. Furthermore, they found that the risk of progression from normal CP to consistent CP, indeterminate CP, and pseudo-CP increased with an increasing ethanol intake. Although alcohol intake is considered a factor that exacerbates chronic PD, the daily alcohol intake of the patients in this study was less than 20 g/day, which may be insufficient to have a significant impact. Other factors that may exacerbate chronic PD include dysfunction of accessory papillae and dietary fat loads, and recent studies have investigated genetic predisposition [32].

Notably, the prognosis of ECP remains unclear. Whitcomb et al. [33] showed that while many studies recognized the existence of an early stage in the progression of chronic pancreatitis, they also highlighted that the signs and symptoms of early disease are nonspecific, progression remains uncertain with an imaging-based approach, and the diagnostic criteria for a definitive diagnosis of chronic pancreatitis using imaging techniques alone are not satisfied.

Asymptomatic patients with PD who were examined in this study lacked clinical symptoms and did not meet the clinical diagnostic criteria for ECP. However, in this study, EUS findings consistent with ECP were observed in 8 of the 17 patients, which suggests that asymptomatic patients with PD may already have changes related to chronic pancreatitis.

Nevertheless, this study is a single-center, retrospective analysis with a small sample size, which limits the generalizability of the findings. The diagnosis of early chronic pancreatitis was based solely on imaging criteria without histopathological confirmation, which may have introduced variability in the interpretation.

## 5. Conclusions

A considerable proportion of asymptomatic patients with PD may exhibit EUS findings suggestive of ECP. These findings indicate that early changes in chronic pancreatitis may be accompanied by asymptomatic PD.

## Figures and Tables

**Table 1:** Characteristics of the asymptomatic pancreas divisum patients (*n* = 17).

Sex	9 Males (53%), 8 Females (47%)
Age (years)	median 68, range 52–82
Main pancreatic duct (mm)	median 2.5, range 0.9–3.3
Alcohol users	8 (47%)

**Table 2:** Endoscopic ultrasonography findings in asymptomatic pancreas divisum patients according to the Rosemont Classification.

Parenchymal criteria		
hyperechoic foci with shadowing	major A	0 (0%)
hyperechoic foci without shadowing	minor	9 (53%)
stranding	minor	11 (65%)
lobularity with honeycombing	major B	0 (0%)
lobularity without honeycombing	minor	3 (18%)
hyperechoic main pancreatic duct margin	minor	5 (29%)
Duct criteria		
dilated side branches	minor	7 (41%)
cyst	minor	6 (35%)
main pancreatic duct dilatation	minor	0 (0%)
main pancreatic duct calculi	major A	0 (0%)
irregular main pancreatic duct contour	minor	0 (0%)
Endoscopic ultrasonography diagnosis of chronic pancreatitis based on consensus criteria		
consistent with chronic pancreatitis		0 (0%)
suggestive of chronic pancreatitis		3 (18%)
indeterminate for chronic pancreatitis		5 (29%)
normal		9 (53%)

**Table 3:** Endoscopic ultrasonography findings and diagnosis of early chronic pancreatitis according to the Japanese Diagnostic Criteria for Early Chronic Pancreatitis 2019 in asymptomatic pancreas divisum patients.

	*n*
Hyperechoic foci/stranding	14 (82%)
Lobularity	3 (18%)
Hyperechoic main pancreatic duct margin	5 (29%)
Dilated side branches	7 (41%)
Endoscopic ultrasonography diagnosis of early chronic pancreatitis based on the Japanese Diagnostic Criteria for Early Chronic Pancreatitis 2019	8 (47%)

**Table 4:** Comparison of background factors with or without endoscopic ultrasonography findings of early chronic pancreatitis.

	ECP (+)	ECP (−)	*p*
*n*	8	9	
Sex	5 males (63%), 3 females (37%)	4 males (44%), 5 females (56%)	0.64
Age (years)	median 63, IQR 52–78	median 59, IQR 49–82	0.70
MPD (mm)	median 2.15, IQR 0.9–3.3	median 2.2, IQR 1.6–3.1	0.70
Alcohol users (*n*)	4 (50%)	4 (44%)	1.00

ECP, early chronic pancreatitis; IQR, interquartile range; MPD, main pancreatic duct.

**Table 5:** Comparison of background factors and endoscopic ultrasonography findings according to the RC with or without alcohol intake.

	Alcohol (−)	Alcohol (+)	*p*
*n*	9	8	
Sex (male), *n*	4 (44%)	5 (63%)	0.64
Age (years), median (IQR)	71 (55–82)	63 (49–77)	0.29
MPD (mm), median (IQR)	2.3 (1.2–3.3)	2.6 (0.9–3.0)	0.50
Major/minor criteria, median (IQR)			
Minor	2 (1–5)	2.5 (1–5)	0.62
Major A	0	0	
Major B	0	0	
EUS diagnosis of CP based on consensus criteria			
Suggestive	2	1	1.00
Indeterminate	2	3	0.62
Total	4	4	1.00

CP, chronic pancreatitis; EUS, endoscopic ultrasonography; IQR, interquartile range.

## Data Availability

The raw data supporting the conclusions of this article will be made available by the authors on request.
